# Multi-Component Intervention Program on Habitual Physical Activity Parameters and Cognitive Function in Patients with Mild Cognitive Impairment: A Randomized Controlled Trial

**DOI:** 10.3390/ijerph18126240

**Published:** 2021-06-09

**Authors:** Min-Ki Jeong, Kyung-Won Park, Je-Kwang Ryu, Gwon-Min Kim, Hyun-Hun Jung, Hyuntae Park

**Affiliations:** 1 College of Arts and Sports, Dong-A University, Busan 49315, Korea; prof.mkjeong@gmail.com (M.-K.J.); jhh320@gmail.com (H.-H.J.); 2 Busan Metropolitan Dementia Center, Busan 49201, Korea; neuropark@dau.ac.kr; 3 Department of Neurology, College of Medicine, Dong-A University, Busan 49201, Korea; 4 Department of Health Sciences, Graduate School, Dong-A University, Busan 49315, Korea; ljk080617@gmail.com; 5 Health Convergence Medicine Laboratory, Biomedical Research Institute, Pusan National University Hospital, Busan 49241, Korea; rlarnjsals47@gmail.com; 6 Center for Gerontology and Social Science, National Center for Geriatrics and Gerontology, Obu 474-8511, Japan

**Keywords:** multi-component intervention, mild cognitive impairment, dementia, habitual physical activity parameters, cognitive function

## Abstract

Age-related dementia refers to a state in which someone experiences multiple cognitive function impairment due to degenerative brain disease, and which causes difficulties in their daily life or social life. Dementia is the most common and serious obstacle in later life. Early intervention in the case of patients who are in the mild cognitive impairment (MCI) stage among the high-risk group can maintain and improve their cognitive function. The purpose of the current trial is aimed at investigating the association between a multi-component (exercise with cognitive) intervention program and habitual physical activity parameters on cognitive functions in MCI patients. Neuropsychological cognitive and depression assessments were performed by neuropsychologists according to normalized methods, including the Korean mini-mental State examination (K-MMSE) and modified Alzheimer’s disease assessment scale-cognitive subscale (ADAS-Cog) and cognitive assessment tool (attention, processing speed), and the Korean version of the geriatric depression scale (SGDS-K), both at baseline and at a 12 weeks follow-up. The 12-week multi-component intervention improved cognitive function and habitual physical activity parameters in patients with MCI relative to controls. A multi-component intervention program for patients with MCI is considered to be an effective method of dementia prevention by improving global (ADAS-Cog) and frontal (trail-making test, digit symbol substitution test) cognition and habitual physical activity parameters such as moderate to vigorous physical activity and step count. In addition, it is important to encourage habitual physical activities to ensure that exercise intervention strategies are carried out at the duration and intensity required for improving physical and cognitive wellbeing and obtaining health benefits.

## 1. Introduction

Age-related dementia refers to a state in which multiple cognitive function impairments due to degenerative brain disease cause difficulties in daily life or social life [1]. Therefore, it is important to develop prevention and treatment methods, as the occurrence of dementia requires enormous medical and care costs. Currently, a pharmacological therapy method associated with dementia is used, but curable treatment is difficult [2]. Early intervention of patients who are in the mild cognitive impairment (MCI) stage among the high-risk group can maintain and improve cognitive function [3].

Previous research on exercise and cognitive function reported that people who had done much exercise during their youth or who had participated in regular exercise could decrease the risk of cognitive impairment and delay decreases in cognitive function compared to those who did not exercise [2,4,5,6]. Lautenschlager et al. [7] reported that a moderate amount of exercise of 150 min per week delayed the decrease of cognitive function due to aging. Randomized controlled trials of the effects of exercise on MCI elderly people and cognitive function effect [8,9] reported that aerobic exercise increased brain volume in domains associated with cognitive function decline by aging [10,11]. Also, Erickson et al. [12] suggested that exercise is effective in protecting the hippocampus and preventing cognitive function decrease by reporting a positive correlation between hippocampal volume and aerobic ability and spatial memory.

However, a previous Nicola et al. [13] meta-analysis reported that there is very limited evidence that single bout type exercise improves the cognitive function of individuals with MCI. Also, when only physical activity (PA) was applied, the cognitive function showed little difference in large scale longitudinal research compared to education groups [14]. Meanwhile, to solve this problem, extensive application of exercise and cognitive intervention has been suggested [15,16]. Holtzer et al. [17] reported that multi-task exercise, preferable to single-bout type exercise, is effective in improving brain function. The multi-component is the execution of other tasks with a single task, or two or more tasks are performed at the same time [18]. Since the multi-task exercise method with cognitive and exercise task, rather than just one exercise method, is more effective at improving cognitive function [19,20], it can be predicted that it impacts the brain’s cognitive reserve. However, there is a lack of studies on cognitive function improvement in programs that combine exercise with multi-component cognitive intervention in patients with MCI in South Korea. Furthermore, previous research is limited to community-based settings for elderly people, which may be limited by the lack of specialists to perform physical and cognitive function assessments, such as in a clinical setting. Moreover, the quantity of studies on the relationship between PA parameters that combine exercise and cognitive tasks and cognitive and brain function for patients with MCI is limited.

The purpose of the current trial is aimed at investigating the association between a multi-component intervention program and habitual PA parameters on cognitive functions in MCI patients.

## 2. Materials and Methods

### 2.1. Participants

**Figure 1:**
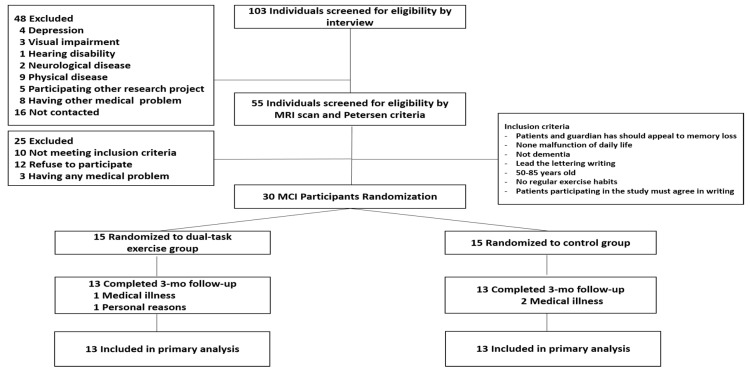
Flow diagram of participants and outcomes.

The sample size was estimated using G*Power software (Version 3.1.9.2) [21,22]. The sample size was determined using a priori power calculations (repeated measures, within-between interaction, power of 0.80, 2-sided α-error, 2 groups, 2 measurements) based on the primary outcome of the ADAS-Cog from a comparable study [23]. The required sample size calculated with an additional 15% increase per group, taking into account drop outs, was 15 participants per group. The MCI diagnosis [24] was based on medical evaluations through a clinical interview by a dementia specialist. The diagnosis was also based on neurological examinations, magnetic resonance imaging, and detailed neuropsychological assessments. Non-amnestic MCI was also excluded from the subtypes of MCI classified by the Petersen criteria [25]. A total of thirty patients with MCI were enrolled. According to random assignment, the subjects were divided into a multi-component intervention group and a control group (n = 15 each). Four of them were excluded from the study for personal reasons (n = 1) and medical illness (n = 3). The final study sample comprised a multi-component intervention group and a control group (n = 13 each), 26 subjects in total Figure 1. The participants’ demographic and physical characteristics are shown in Table 1. All participants signed written consent forms prior to participating in the protocol, and the study was approved by the Institutional Review Board of Dong-A University (2-104709-AB-N-01-2017-HR-058-02).

### 2.2. Measurement of Body Composition

Before the intervention and after 12 weeks, trials were performed in the morning to exclude diurnal variation. Body composition was assessed using the N20 (N20, AIIA Communication Inc., Seongnam-si, Korea), which measured height, weight, body fat percentage, fat mass, lean body mass (LBM), and appendicular skeletal muscle mass (ASM) before and after 12 weeks. Body mass index (BMI) was calculated as weight (kg) divided by the square of the height (m) and appendicular skeletal muscle mass index (ASMI) was calculated as ASMI (kg) divided by the square of the height (m). Also, waist and hip circumference were measured with a tapeline (Martine’s body measuring instrument), and waist-to-hip ratio (WHR) was calculated as waist divided by the hip circumference.

### 2.3. Habitual Physical Activity Measurement

Habitual PA parameters were measured using the Lifecoder (Kenz lifecoder, Tokyo, Japan). An accelerometer’s activity intensity degree (i.e., 11 level: 0, 0.5, 1~9; higher number means high intensity) was measured every four seconds during each day for twelve weeks, and the metrics of PA measured the intensity (metabolic equivalents: METs) [26] and step count (step/day) [27] of PA (moderate to vigorous physical activity: MVPA) from moderate intensity (3–6 METs) to high intensity (>6 METS), but not low intensity (<3 METs). Details of the process were described in previous studies [26,28,29,30,31,32].

### 2.4. Neuropsychological Cognitive and Depression Assessment

Neuropsychological cognitive assessments were performed by neuropsychologists according to normalized methods, which include the modified Alzheimer’s disease (AD) assessment scale-cognitive subscale (ADAS-Cog, range 0–89) [33] and the Korean mini-mental state examination (K-MMSE) [34,35]. In addition, attention (trail making test part A, B: TMT-A; range 0–90 s.) and processing speed (digit symbol substitution test: DSST; range 0–90 pt.) were measured for the prefrontal lobe cognitive function by using an iPad (Apple, Cupertino, CA, USA) cognitive assessment tool developed by the National Center for Geriatrics and Gerontology (NCGG) [36]. Symptoms of depression were assessed by GDS [37], and using the Korean version of the geriatric depression scale (SGDS-K) [38]. Each question awards 0 or 1 point, and a higher scores mean a higher level of depression, with 10 points being suggested as an optimal score to indicate depression.

### 2.5. Multi-Component Intervention Program

The multi-component intervention program involved 12 twice-weekly 90 min sessions focused on physical and cognitive activities that combined aerobic exercise, PA promotion and behavior modification, and cognitive and exercise multi-task programs [23,39,40,41]. Between five and six people participated in each session conducted by two geriatric exercise specialists and one occupational therapist or nurse. Each session featured, in order, a warm-up for 10 min, main exercise for 50 min (aerobic and cognitive intervention training), cooling down for 10 min, and promoting habitual PA education and feedback for 20 min. Before and after the intervention, shuttle walking and stretching were mainly conducted, and vital checks such as blood pressure were carried out between the main exercise (walk in line, toe rock-paper-scissors, step box, thera-band exercise, body rock-paper-scissors, ladder, etc.) and break time. In the multi-component intervention program, participants conducted cognitive tasks (such as speaking, counting, word games, performing fast uncomplicated numerical calculations, and playing a simple memory span game) while doing exercise. The control group was required to maintain daily life and participated in the monthly educational class. Monthly health management education on the importance of health care, nutritional habits for health, and the benefits of exercise was conducted by a specialist (nutritionist and social worker) once every four weeks (weeks 3, 7, and 11), and body composition checks and personal consultations were conducted at intervals of two weeks (weeks 1, 5, and 9).

Meanwhile, in order to enhance the amount of PA beside the intervention program, the study provided an exercise training program manual that can be done in daily life and confirmed the person’s activity levels in each subject to evaluate weekly PA by using an accelerometer. In addition, the heart rate was self-assessed instantly following the multi-component intervention based on pulse rate using the portable wireless heart rate meter Polar S410s (Polar Electro Co., Kempele, Finland). The conditions for exercise intensity were 40~50% of heart rate reserve in weeks 1~3, 50~65% in weeks 4~6, and 60~80% in weeks 7~12.

### 2.6. Statistical Analysis

All data statistical computations and management were performed using the IBM SPSS Statistics version 23.0 software package (SPSS Inc., Chicago, IL, USA), and descriptive statistics of all variables were reported as means ± standard deviations. The baseline characteristics were used to conduct an independent t-test for continuous data and the Chi-square test and Fisher’s exact test for categorical data. A two-way repeated measures ANOVA was performed for the dependent (outcome) variables to determine whether the interventions produced the within-between group and interactive group × time effects, followed by post-hoc tests of the variables. In the case of statistically significant interaction effects, paired *t*-tests for differences between baseline and after intervention within group and independent *t*-tests for differences between the two groups over time were conducted. Statistical significance was accepted at the *p* < 0.05 level.

## 3. Results

### 3.1. Body Composition and Habitual Physical Activity Parameters

The changes in body composition and habitual PA parameter are shown in Table 2. In the multi-component intervention group, LBM (*p* < 0.01), muscle mass (*p* < 0.01), lower limb muscle mass (*p* < 0.01), MVPA (*p* < 0.01), and step count (*p* < 0.01) increased validly and hip circumference (*p* < 0.001) decreased validly. Also, there was a significant difference in the interaction between the group and time in MVPA (*p* = 0.04) and step count (*p* = 0.01).

### 3.2. Cognitive Function and Depression

Table 3 shows the changes in cognitive function and depression. In the multi-component intervention group, SGDS-K (*p* < 0.05), modified ADAS-cog (*p* < 0.05), mean TMT-A (*p* < 0.01), mean TMT-B (*p* < 0.01) significantly decreased, while mean DSST (*p* < 0.01) significantly increased. Also, a significant difference in the interaction between the group and time was shown in SGDS-K (*p* = 0.01), mean TMT-A (*p* < 0.05), mean TMT-B (*p* = 0.01), and mean DSST (*p* = 0.02).

## 4. Discussion

The purpose of the current trial is to investigate the relationship between a multi-component intervention program and habitual PA parameters and cognitive function in MCI patients. There was a significant improvement in body composition such as LBM, muscle mass, and lower limb muscle mass between pre and after 12 week multi-component intervention. However, no significant interaction between group and time was found. The multi-component intervention group showed greater changes than the control group, suggesting that research on the long-term effect of this treatment on body composition change is required. The daily PA change, MVPA, and step count showed significant differences in interaction between group and time. Lautenschlager et al. [7] reported that moderate exercise of 150 min per week led to a delayed reduction in cognitive function due to aging. A cross-sectional study of elderly women aged 65 years or older [6] reported that those who exercise regularly when they are young are likely to experience a decreased risk of cognitive impairment and are more likely to see a delay in decreased cognitive function compared to those who do not exercise regularly when they are young. Considering that PA is significantly correlated with cognitive function, the increase in PA is expected to have a positive effect on the activity of daily living (ADL) and social activity. In addition, previous studies reported that MVPA rather than low intensity was effective in increasing hippocampus volume and memory [11,42]. Considering the results of previous studies, it is thought that the increase in MVPA in this study had a positive effect on improving hippocampus volume and memory. These findings suggest that MVPA improves cognitive function. Many elderly people experience a reduced quality of life due to the decline of physical function, cognitive impairment, depression, and uneasiness [43]. Wilson et al. [44] reported that elderly people suffering from depression are subjected to cognitive function decline approximately 20% faster than those who do not have such impairment, suggesting a correlation between depression symptoms and cognitive function. In a study of relationships between exercise and depression, Lindwall et al. [45] reported that active elderly people enjoy lower levels of depression than their inactive counterparts, and that elderly people who exercise regularly showed a significantly lower level of depression than those who do not. These findings suggest that exercise contributes to improving depression. The present study indicates that depression score was significantly improved among the multi-component intervention group and a significant difference in the group and time interaction was found, indicating that exercise had a positive effect on alleviating depression levels. This finding is consistent with Sjosten et al.’s [46], who found that exercise improves depression. However, the positive effect that exercise has on depression tends to decrease over time. Therefore, as prior works [47,48,49] have suggested, future researchers need to track those who stop exercise and investigate the effect of stopping exercise on the change in depression level.

Jacobs et al. [50] found that executive function and attention among MCI patients were significant predictors of Alzheimer’s disease, and MCI patients displayed declined neurocognitive functions such as memory, executive function, attention, and exercise function compared to normal elderly people [25]. In their research on exercise and cognitive function, Yaffe et al. [51] reported that elderly women aged 65 or older who exercise regularly displayed delayed decrease of cognitive function. In their meta-analysis of MCI and dementia in elderly people, Heyn et al. [52] indicated that exercise, regardless of its type, is positively correlated with cognitive function as well as physical health, suggesting that exercise contributes to the improvement of cognitive function.

In the changes in cognitive function, there was a significant difference in the interaction between the group and time in mean TMT-A, TMT-B, and mean DSST. This finding is similar to those of multifaceted exercise intervention program studies [17,18,19,20] in community-based settings. Specifically, considering that the prevention of depression and decreased cognitive functions are important for dementia prevention, the multi-component intervention effect in a clinical setting is considered to be meaningful. Follow-up studies are needed to test the program effect after the program is stopped. Therefore, a multi-component intervention program has a positive effect on the prevention of depression and impaired cognitive functions. In particular, the increase of PA may have a positive effect on the frontal lobe that is related to cognitive function. Although the improvement of cognitive function through multi-component intervention is the meaningful result, maintaining the habit of regular exercise and PA can be important factors for the prevention of dementia.

This research has some limitations. First, the accuracy of these data is limited by the small sample size. Moreover, although nutritional intake such as protein does affect the subject’s physical function, we were unable to investigate nutrition. Therefore, further study is required in the form of large randomized controlled trials that investigate the synergist effect that exercise and protein have on this population.

## 5. Conclusions

A multi-component intervention program for patients with MCI is a potentially effective method of dementia prevention by improving cognitive function and habitual PA parameters, and it is important to encourage habitual physical activities to ensure that exercise intervention strategies are carried out at the duration and intensity required for improving physical and cognitive ability and obtaining health benefits.

## Figures and Tables

**Table 1:** Baseline characteristics of demographic and physical characteristics, habitual physical activity parameter, depression, and cognitive function.

Variable	Intervention Group (n = 13)	Control Group (n = 13)	*p*-Value
**Demographic**			
Age (years)	70.23 ± 7.47	71.77 ± 5.53	0.73
Male, n (%)	4(30.8%)	4(30.8%)	1.00
Educational level (years)	7.23 ± 4.04	7.38 ± 3.33	0.65
Job sequences, n (%)	1(7.7%)	1(7.7%)	1.00
Smoking (yes), n (%)	0(0%)	1(7.7%)	1.00
Alcohol consumption (yes), n (%)	4(30.8%)	2(15.4%)	0.65
**Physical characteristics**			
Height (m)	1.56 ± 0.09	1.55 ± 0.08	0.42
Weight (kg)	55.98 ± 7.48	55.85 ± 5.53	0.29
Body mass index (kg/m^2^)	23.05 ± 2.16	23.18 ± 1.89	0.37
Waist hip ratio	0.86 ± 0.05	0.88 ± 0.05	0.72
Fat (%)	31.58 ± 11.40	30.54 ± 6.71	0.17
Lean body mass (kg)	38.58 ± 9.10	38.72 ± 5.70	0.11
ASMI (kg/m^2^)	6.36 ± 1.38	6.72 ± 1.27	0.49
**Habitual physical activity parameters**			
MVPA (min/day)	23.83 ± 19.09	18.61 ± 16.07	0.60
Step count (steps/day)	7543 ± 3150	6592 ± 2961	0.87
**Depression and Cognitive function**			
SGDS-K (score)	5.54 ± 4.27	4.23 ± 3.35	0.09
K-MMSE (score)	25.77 ± 2.31	25.00 ± 2.58	0.86
Modified ADAS-cog (score)	24.08 ± 7.75	28.54 ± 8.41	0.65

Variables are means ± SD. ASMI: appendicular skeletal muscle mass index; MVPA: moderate to vigorous physical activity; SGDS-K: Korean version of geriatric depression scale-short form; K-MMSE: Korean version of mini mental state examination; ADAS-cog: Alzheimer`s disease assessment scale-cognitive.

**Table 2:** The changes in body composition and habitual physical activity parameters between the groups at baseline and after 12 weeks.

Variable	Group	Baseline	12 Weeks	% Diff	*p*-Value (Interaction)
**Body Composition**					
Weight (kg)	Intervention	55.98 ± 7.48	56.86 ± 7.39	1.57	0.59
	Control	55.85 ± 5.53	56.30 ± 6.45	0.81	
Body mass index (kg/m^2^)	Intervention	23.05 ± 2.16	23.35 ± 2.19	1.30	0.87
	Control	23.18 ± 1.89	23.52 ± 1.86	1.47	
Waist circumference (cm)	Intervention	83.03 ± 7.14	79.15 ± 19.01	−4.67	0.26
	Control	84.08 ± 7.18	85.54 ± 6.76	1.74	
Hip circumference (cm)	Intervention	97.04 ± 4.78	95.23 ± 5.23	−1.87 *	0.76
	Control	95.94 ± 4.45	93.62 ± 5.16	−2.42	
Waist hip ratio	Intervention	0.86 ± 0.05	0.83 ± 0.19	−3.49	0.24
	Control	0.88 ± 0.05	0.92 ± 0.09	4.55	
Fat (%)	Intervention	31.58 ± 11.40	29.45 ± 10.31	−6.75	0.68
	Control	30.54 ± 6.71	27.53 ± 9.06	−9.86	
Fat mass (kg)	Intervention	17.39 ± 5.68	16.60 ± 5.66	−4.54	0.64
	Control	16.97 ± 3.95	15.60 ± 5.39	−8.07	
Lean body mass (kg)	Intervention	38.58 ± 9.10	40.41 ± 8.62	4.74 *	0.88
	Control	38.72 ± 5.70	40.68 ± 6.17	5.06 *	
Muscle mass (kg)	Intervention	35.98 ± 8.49	37.66 ± 8.05	4.67 *	0.87
	Control	36.09 ± 5.32	37.92 ± 5.74	5.07 *	
Lower limb muscle mass (kg)	Intervention	11.72 ± 3.41	12.28 ± 3.27	4.78 *	0.28
	Control	11.52 ± 2.28	11.77 ± 2.15	2.17	
ASMI (kg/m^2^)	Intervention	6.36 ± 1.38	6.56 ± 1.28	3.15	0.86
	Control	6.72 ± 1.27	6.87 ± 0.74	2.23	
**Habitual Physical Activity Parameters**					
MVPA (min/day)	Intervention	23.83 ± 19.09	32.57 ± 21.52	36.68 *	0.04
	Control	18.61 ± 16.07	19.86 ± 22.10	6.72	
Step count (steps/day)	Intervention	7543 ± 3150	10189 ± 3768	35.08 *	0.01
	Control	6592 ± 2961	6342 ± 3732	−3.79	

Variables are means ± SD. ASMI: appendicular skeletal muscle mass index, MVPA: moderate to vigorous physical activity. Significantly different from baseline compared to 12 weeks: * *p* < 0.05.

**Table 3:** The changes in depression and cognitive function between the groups at baseline and after 12 weeks.

Variable	Group	Baseline	12 Weeks	% Diff	*p*-Value (Interaction)
SGDS-K (score)	Intervention	5.54 ± 4.27	4.38 ± 4.01	−20.94 *	0.01
	Control	4.23 ± 3.35	6.31 ± 4.44	49.17 *	
K-MMSE (score)	Intervention	25.77 ± 2.31	25.69 ± 2.53	−0.31	0.72
	Control	25.00 ± 2.58	24.62 ± 2.18	−1.52	
Modified ADAS-cog (score)	Intervention	24.08 ± 7.75	22.23 ± 8.60	−7.68 *	0.11
	Control	28.54 ± 8.41	29.00 ± 7.90	1.61	
Mean TMT-A (s/letter)	Intervention	2.74 ± 1.26	2.01 ± 0.95	−26.64 *	<0.05
	Control	2.19 ± 0.95	2.42 ± 1.41	10.50 *	
Mean TMT-B (s/letter)	Intervention	3.80 ± 1.46	2.40 ± 1.19	−36.84 *	0.01
	Control	2.79 ± 0.91	3.17 ± 1.25	13.62 *	
Mean DSST	Intervention	30.67 ± 3.10	37.08 ± 3.45	20.90 *	0.02
	Control	29.62 ± 3.98	28.46 ± 3.96	−3.92	

Variables are means ± SD. SGDS-K: Korean version of geriatric depression scale-short form; K-MMSE: Korean version of mini mental state examination; ADAS-cog: Alzheimer`s disease assessment scale-cognitive; TMT: trail-making test; DSST: digit symbol substitution test. Significantly different from baseline compared to 12 weeks: * *p* < 0.05.

## Data Availability

Qualified researchers can obtain the data from the corresponding author (htpark@dau.ac.kr). The data are not publicly available due to privacy concerns imposed by the IRB.
